# Supplementary material for: Valence and salience encoding in the central amygdala
Source: eLife. 2025 Jan 10;13:RP101980. doi: 10.7554/eLife.101980 (PMC11723578; doi:10.7554/eLife.101980)
Supplement: Supplementary file 1. [file elife-101980-supp1.docx]

**Supplementary File 1. Results of statistical analyses.**

| **Figure** | | **Test** | **N** | **Statistics** | **P** |
| --- | --- | --- | --- | --- | --- |
| **Main Figure** | | | | | |
| 1 | C  left | One-way repeated-measures ANOVA | 10 | F(3.392,30.53)=7.422  Main effects = Days | P=0.0005 |
|  | C  middle | One-way repeated-measures ANOVA | 10 | F(2.521, 22.69)=20.41  Main effects = Days | P<0.0001 |
|  | C  right | One-way repeated-measures ANOVA | 10 | F(1.270, 11.43)=14.69  Main effects = Days | P=0.0017 |
|  | D | One-way repeated-measures ANOVA | 10 | F(3.997, 35.97)=13.49  Main effects = Trials | P<0.0001 |
| 3 | B | Chi-square | Day 1  (148,104,826)  Day 10 (307,229,336) | X^2^ (2) = 290.6 | P<0.0001 |
|  | C | Unpaired  t-test | Day1=147  Day10=307 | t(452)=3.474  95% CI: 42.09 to 151.8 | P=0.0006 |
|  | D | Unpaired  t-test | Day1=104  Day10=229 | t(331)=1.309  95% CI: -1.497 to 7.416 | P=0.1915 |
| 5 | C  Both excited | Paired  t-test | 36 | t(35)=1.025  95% CI: -155.6 to 51.20 | P=0.3124 |
|  | C  Both inhibited | Paired  t-test | 10 | t(9)=0.3386  95% CI: -30.00 to 40.57 | P=0.7427 |
|  | D  Food↑ -  Shock↓ | Paired  t-test | 16 | t(15)=4.025  95% CI: -812.9 to -250.1 | P=0.0011 |
|  | D  Food↓ -  Shock↑ | Paired  t-test | 25 | t(24)=7.592  95% CI: 190.3 to 332.4 | P<0.0001 |
|  | E  Food↑ -  nonShock | Paired  t-test | 60 | t(59)=5.806  95% CI: -425.2 to -207.2 | P<0.0001 |
|  | E  Food↓ -  nonShock | Paired  t-test | 50 | t(49)=11.44  95% CI: 61.31 to 87.45 | P<0.0001 |
|  | E  nonFood - Shock↑ | Paired  t-test | 34 | t(33)=4.118  95% CI: 92.87 to 274.2 | P=0.0002 |
|  | E  nonFood - Shock↓ | Paired  t-test | 13 | t(12)=2.046  95% CI: -109.1 to 3.423 | P=0.0633 |
| **Figure supplement** | | | | | |
| S3-1 | C | Paired  t-test | 62 | t(61)=1.538  95% CI: -43.03 to 5.617 | P=0.0646 |
|  | D | Paired  t-test | 50 | t(49)=2.510  95% CI: 6.027 to 54.46 | P=0.0077 |
|  | E | Paired  t-test | 345 | t(344)=1.514  95% CI: -67.00 to 8.707 | P=0.0654 |
|  | F | Paired  t-test | 214 | t(213)=3.787  95% CI: -15.35 to -4.840 | P<0.0001 |
| S4-1 | C | Paired  t-test | 19 | t(18)=0.2655  95% CI: -36.93 to 47.62 | P=0.3968 |
|  | D | Paired  t-test | 24 | t(23)=0.6971  95% CI: -27.45 to 13.61 | P=0.2464 |
|  | E | Paired  t-test | 157 | t(156)=5.246  95% CI: -116.1 to -52.60 | P<0.0001 |
|  | F | Paired  t-test | 62 | t(61)=0.7995  95% CI: -5.481 to 12.79 | P=0.2135 |
| S5-1 | A | Unpaired  t-test | Food=345  Shock=157 | t(500)=21.76  95% CI: -7.816 ± 0.3591 | P<0.0001 |
|  | B | Unpaired  t-test | Food=345  Shock=157 | t(500)=3.210  95% CI: -4.265 to -1.026 | P=0.0014 |
|  | G | Paired  t-test | 10 | t(9)=0.6739  95% CI: -8.572 to 4.637 | P=0.5173 |
|  | J | Paired  t-test | 10 | t(9)=0.8884  95% CI: -5.427 to 12.45 | P=0.3974 |
| S5-3 | I  food_  excited | Unpaired  t-test | Food before Shock=61  Food after Shock=51 | t(110)=0.6962  95% CI: -198.0 to 95.05 | P=0.4877 |
|  | I  food_  inhibited | Unpaired  t-test | Food before Shock=51  Food after Shock=34 | t(83)=1.162  95% CI: -5.664 to 21.57 | P=0.2486 |
|  | J  shock_  excited | Unpaired  t-test | Shock before Food=54  Shock after Food=41 | t(93)=1.435  95% CI: -164.0 to 26.41 | P=0.1547 |
|  | J  shock_  inhibited | Unpaired  t-test | Shock before Food=17  Shock after Food=22 | t(37)=0.8996  95% CI: -44.26 to 17.04 | P=0.3741 |
|  | K  CS^Food^-  excited | Unpaired  t-test | CS^Food^ before Shock=17  CS^Food^ after Shock=9 | t(24)=1.492  95% CI: -31.34 to 5.038 | P=0.1487 |
|  | K  CS^Food^-inhibited | Unpaired  t-test | CS^Food^ before Shock=7  CS^Food^ after Shock=8 | t(13)=1.290  95% CI: -24.76 to 6.244 | P=0.2195 |
|  | L  CS^Shock^-excited | Unpaired  t-test | CS^Shock^ before Food=9  CS^Shock^ after Food=4 | t(11)=1.069  95% CI: -71.03 to 24.57 | P=0.3078 |
|  | L  CS^Shock^-inhibited | Unpaired  t-test | CS^Shock^ before Food=8  CS^Shock^ after Food=10 | t(16)=0.4894  95% CI: -10.41 to 16.67 | P=0.6312 |
